# Alternative-NHEJ Is a Mechanistically Distinct Pathway of Mammalian Chromosome Break Repair

**DOI:** 10.1371/journal.pgen.1000110

**Published:** 2008-06-27

**Authors:** Nicole Bennardo, Anita Cheng, Nick Huang, Jeremy M. Stark

**Affiliations:** 1 Department of Radiation Biology, Beckman Research Institute of the City of Hope, Duarte, California, United States of America; 2 City of Hope Graduate School of Biological Sciences, Duarte, California, United States of America; Brandeis University, United States of America

## Abstract

Characterizing the functional overlap and mutagenic potential of different pathways of chromosomal double-strand break (DSB) repair is important to understand how mutations arise during cancer development and treatment. To this end, we have compared the role of individual factors in three different pathways of mammalian DSB repair: alternative-nonhomologous end joining (alt-NHEJ), single-strand annealing (SSA), and homology directed repair (HDR/GC). Considering early steps of repair, we found that the DSB end-processing factors KU and CtIP affect all three pathways similarly, in that repair is suppressed by KU and promoted by CtIP. In contrast, both KU and CtIP appear dispensable for the absolute level of total-NHEJ between two tandem I-SceI–induced DSBs. During later steps of repair, we find that while the annealing and processing factors RAD52 and ERCC1 are important to promote SSA, both HDR/GC and alt-NHEJ are significantly less dependent upon these factors. As well, while disruption of RAD51 causes a decrease in HDR/GC and an increase in SSA, inhibition of this factor did not affect alt-NHEJ. These results suggest that the regulation of DSB end-processing via KU/CtIP is a common step during alt-NHEJ, SSA, and HDR/GC. However, at later steps of repair, alt-NHEJ is a mechanistically distinct pathway of DSB repair, and thus may play a unique role in mutagenesis during cancer development and therapy.

## Author Summary

Changes to the sequence of DNA, or mutations, can disrupt cellular growth control genes, which can lead to cancer development. Such mutations likely arise from damage to DNA that is repaired in a way that fails to restore the original sequence. One type of DNA damage is a chromosomal double-strand break. We have developed assays to measure how these breaks are repaired, and also how such repair can lead to mutations. In particular, we present an assay to measure a pathway of repair that results in deletion mutations, often with evidence of short homologous sequences at the repair junctions (alt-NHEJ). We have compared the genetic requirements of this repair pathway in relation to other pathways of repair that use extensive homology. We find that factors KU and CtIP appear to affect the initial stages of repair of each of these pathways, regardless of the length of homology. However, these pathways appear to diverge at later steps, as relates to the role of the repair factors RAD52, ERCC1, and RAD51. Given that mutations observed in some cancer cells are consistent with alt-NHEJ repair, these mechanistic descriptions provide models for how such mutations could arise in cancer.

## Introduction

Faithful repair of DNA damage is essential to suppress genetic instability and tumorigenesis. Conversely, the efficacy of cancer therapies that utilize DNA damaging agents is likely limited by the ability of cancer cells to repair such damage. One form of DNA damage that is prone to causing mutations is a chromosomal double-strand break (DSB), which can result from DNA replication, reactive oxygen species, radiation therapy, and some types of chemotherapy [1]. Characterizing the factors and pathways of DSB repair is important to understand the process of mutagenesis during cancer development and treatment.

Non-homologous end joining (NHEJ) is a major pathway of DSB repair, in which the ends are ligated without the use of extensive homology. NHEJ appears to comprise both classical-NHEJ and alternative-NHEJ (alt-NHEJ). Classical-NHEJ requires a number of factors important for V(D)J recombination, including the KU70/80 heterodimer (KU), XRCC4, Ligase IV, and DNA-PKcs [2],[3]. Also, classical-NHEJ is predicted to result in minimal processing of the DSB during repair [3],[4]. In contrast, alt-NHEJ appears to be independent of the above factors, and often results in a deletion with microhomology at the repair junction [4]–[12]. Genetic rearrangements consistent with alt-NHEJ have been observed in chromosomal translocations associated with both spontaneous and therapy-related cancer [13], and in reversion mutations of *BRCA2* following DNA damage caused by PARP-inhibition [14]. Thus, alt-NHEJ-derived mutations appear to be associated with cancer development and may result from some cancer therapeutics.

In contrast to the NHEJ pathways, homology-directed repair (HDR/GC) and single-strand annealing (SSA) employ significant degrees of homology [15]. HDR/GC utilizes a homologous template for gene conversion (GC) through strand-invasion and nascent DNA synthesis. HDR/GC is most precise when the identical sister chromatid is used as the template for repair. Thus, factors that are important for HDR/GC might be expected to be genome stabilizing. In contrast to HDR/GC, SSA involves annealing of homologous single strands to bridge the ends of the DSB, resulting in a deletion between the repeats. Such deletions have been observed between homologous segments of ALU elements in germ-line mutations of several tumor suppressor genes [16]. 

It is not clear to what degree alt-NHEJ is mechanistically distinct from SSA or even HDR/GC in mammalian cells**.** We sought to examine this mechanistic distinction by developing an assay for alt-NHEJ repair of a chromosomal DSB, where the predominant repair product is a 35-nucleotide (nt) deletion with 8 nt of microhomology at the repair junction. We have used this assay, along with a novel method for inducible control of the I-SceI endonuclease in stable cell lines, for a comparative genetic analysis of alt-NHEJ, SSA, and HDR/GC. From these studies, we found that alt-NHEJ shares KU/CtIP-mediated regulation of end-processing in common with SSA and HDR/GC, but involves a unique mechanism for completion of repair with regards to the role of ERCC1, RAD52, and RAD51.

## Results

We have sought to understand the genetic relationship between multiple pathways of DSB repair in mammalian cells, since individual repair pathways show a different propensity for mutagenesis. For this, we used a series of chromosome-integrated reporters to monitor the repair of DSBs induced by the I-SceI endonuclease. Each individual reporter is designed such that repair of I-SceI-induced DSBs by a specific pathway restores a *GFP* expression cassette. Such repair can then be scored in individual cells as green fluorescence using flow cytometric analysis (FACS). In each reporter-containing cell line, the generation of GFP+ cells is confirmed to be absolutely dependent upon expression of I-SceI (data not shown).

### Total-NHEJ Results in a Variety of Repair Products

**Figure 1:**
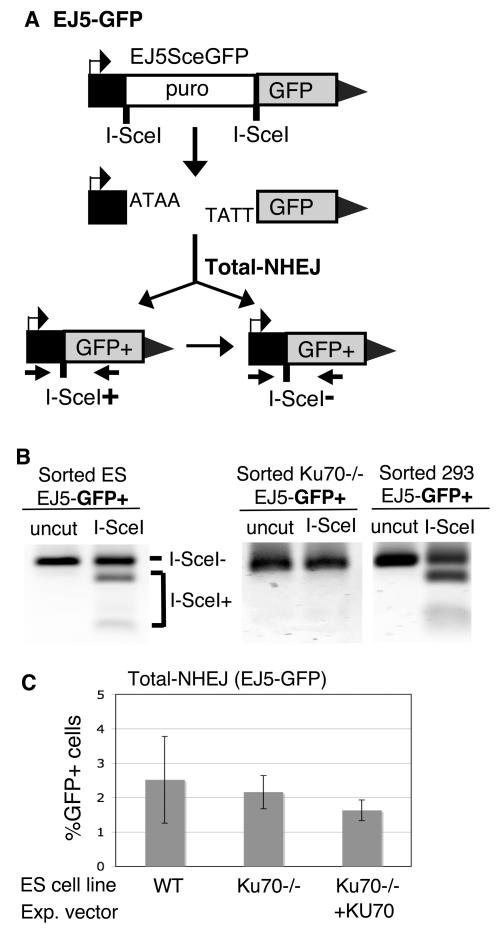
Total-NHEJ repair between two tandem I-SceI sites results in a variety of products. (A) EJ5-GFP is shown along with two classes of NHEJ repair products that can restore a GFP expression cassette: one that restores an I-SceI site (I-SceI+), and one that is I-SceI–resistant (I-SceI-). (B) Restoration of the I-SceI site is common in wild-type cells, but undetectable in KU-deficient cells. EJ5-GFP was integrated into HEK293, wild-type ES, and *Ku70-/-* ES cells. Following transient I-SceI expression in each of these cell lines, GFP+ cells were sorted, and the *GFP* genes were amplified from these samples using primers depicted in (A). Shown are these products digested with I-SceI or left uncut. (C) The overall frequency of total-NHEJ is unaffected by KU deficiency. Shown is the frequency of repair of EJ5-GFP resulting in GFP+ cells from wild-type and *Ku70-/-* ES cells transfected in parallel with an I-SceI expression vector. Also shown are *Ku70-/-* ES cells cotransfected with both I-SceI and KU70 expression vectors.

We have developed two GFP-based chromosomal reporters to measure NHEJ. The first reporter, EJ5-GFP, detects multiple classes of NHEJ events, and thus can be considered an assay for total-NHEJ. We have presented this reporter mostly to provide context for the other reporter (EJ2-GFP), which is designed to monitor only alt-NHEJ events. EJ5-GFP is modeled after other reporters for NHEJ [4],[6],[9], in that it measures repair between two tandem endonuclease cut sites. Specifically, EJ5-GFP contains a promoter that is separated from a *GFP* coding cassette by a *puro* gene that is flanked by two I-SceI sites that are in the same orientation (Figure 1A). Once the *puro* gene is excised by NHEJ repair of the two I-SceI-induced DSBs, the promoter is joined to the rest of the expression cassette, leading to restoration of the *GFP+* gene. Since the two I-SceI-induced DSBs have complementary 3′ overhangs, such NHEJ could potentially restore an I-SceI site. Alternatively, NHEJ could fail to restore the I-SceI site, leading to an I-SceI-resistant site. In addition, a restored I-SceI site could also be re-cleaved and repaired to result in an I-SceI-resistant site.

To determine the relative contribution of these different NHEJ products from repair of EJ5-GFP in mammalian cells, we integrated EJ5-GFP into both wild-type mouse embryonic stem (ES) cells, as well as transformed human embryonic kidney (HEK293) cells (see Materials and Methods). Following transient expression of I-SceI in these cell lines and sorting GFP+ cells, we amplified the *GFP* genes and digested the products with I-SceI. From this analysis, we found evidence of I-SceI-restoration in approximately 40% of the total products from both ES and HEK293 cells (Figure 1B). Regarding the other events (60%), we cloned I-SceI-resistant products from the ES cell line sample and sequenced individual clones (Table S1). Based on these sequences, the I-SceI-resistant NHEJ products showed deletions between 8–27 nucleotides, where the majority of clones (11/12) showed 2–4 nucleotides of microhomology at the junctions. Thus, NHEJ repair of the EJ5-GFP reporter results in either restoration of the I-SceI site, or generation of deletion NHEJ events, often with microhomology at the junctions.

In previous studies with similar NHEJ reporters, KU-deficient cells showed a defect in restoration of the I-SceI site [4],[6]. To test this notion further, we integrated EJ5-GFP into *Ku70-/-* ES cells. We then transfected this line with an I-SceI expression vector, and subsequently measured the frequency of NHEJ events that resulted in a *GFP+* gene, and quantified the restoration of the I-SceI site as described above. In these experiments, we found that *Ku70-/-* ES cells showed approximately equivalent overall frequencies of repair relative to wild-type cells (2.2% and 2.5% respectively, Figure 1C). However, PCR analysis of the repair products in the *Ku70-/-* cells showed only I-SceI-resistant products (Figure 1B). These results suggest that restoration of the I-SceI site during NHEJ repair is absolutely KU-dependent, but that I-SceI-resistant NHEJ events are KU-independent. In summary, EJ5-GFP provides an assessment of total-NHEJ events, which comprises both KU-dependent restoration of an I-SceI site, as well as deletion products with some evidence of microhomology at the junctions.

### Alt-NHEJ Is Suppressed by KU in Mammalian Cells

**Figure 2:**
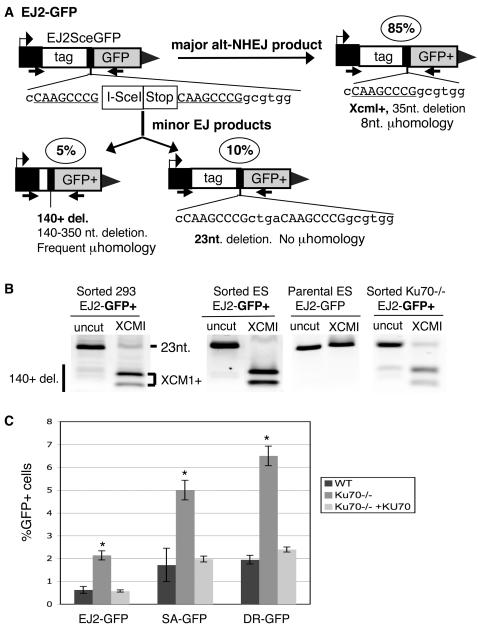
Alt-NHEJ is suppressed by KU. (A) EJ2-GFP is shown with 3 NHEJ products that are found to result in GFP+ cells. Shown in the ovals are the relative contributions of these products, based on the analysis shown in (B). The predominant GFP+ product, labeled Xcm1+, uses 8 nts of homology flanking the I-SceI site to generate an XCM1 site, resulting in a 35 nt deletion. (B) Analysis of EJ2-GFP repair products that restore the *GFP+* gene. EJ2-GFP was integrated into HEK293, wild-type ES, and *Ku70-/-* ES cells. Using the primers shown in (A), the *GFP* genes were amplified from the parental ES EJ2-GFP cell line, and also from sorted GFP+ cells from each of the above cell lines following transient I-SceI expression. Shown are these amplification products, which were either uncut or cut with XCM1. (C) Repair by alt-NHEJ (EJ2-GFP) is suppressed by KU. Shown are the frequencies of alt-NHEJ repair, following transient I-SceI expression, for the wild-type and *Ku70-/-* EJ2-GFP cell lines, along with the *Ku70-/*- line co-transfected with an expression vector for KU70. Also shown are parallel experiments with the SA-GFP and DR-GFP reporters (see Figure 3). Asterisks denote a statistical difference in repair efficiency between *Ku70-/-* versus both wild-type, as well as *Ku70-/-* with transient expression of KU70 (*p*<0.0005).

We have chosen to focus on the subset of total-NHEJ events that show evidence of microhomology at the junctions, also called alt-NHEJ events. For this, we developed a novel reporter (EJ2-GFP), which is designed so the GFP+ products would predominantly reflect a discrete alt-NHEJ event. This reporter contains a single expression cassette for an N-terminal tag (NLS/Zinc-finger, [17]) fused to GFP, except the coding sequence is disrupted between the tag and GFP by an I-SceI site followed by stop codons in all three reading frames (Figure 2A). As well, the I-SceI site and stop codons are flanked by 8 nts. of microhomology, which if annealed during alt-NHEJ would restore the coding frame between the tag and *GFP*, and cause a 35 nt deletion. This alt-NHEJ repair product also generates an XCM1 restriction site.

We determined the contribution of the XCM1+ product relative to total *GFP+* repair products of EJ2-GFP, as integrated in ES and HEK293 cells. For this, we sorted GFP+ cells that resulted from I-SceI expression, amplified the *GFP* genes by PCR, and digested the amplification products with XCM1. From these experiments, we found that the XCM1+ product accounts for approximately 85% of the total repair products in both ES and 293 cells (Figure 2A and 2B). In addition to this predominant repair event, GFP+ products derived from EJ2-GFP also include a few minor repair events, which we identified by sequencing of cloned PCR products (Figure 2A and 2B; see Table S2). For instance, one minor repair event involves a 23 nt deletion with no evidence of microhomology at the junctions (approximately 10% of total events). The final set of events showed larger deletions, which ranged between 140–350 nt and showed microhomology at the repair junctions (approximately 5%). The larger deletion products apparently restore a *GFP+* cassette because the *GFP* start codon was placed proximal to the transcription start site (unpublished data). In summary, while GFP+ products derived from EJ2-GFP can include some minor repair events, the predominant event (XCM1+) involves 8 nt of microhomology and a 35 nt deletion, which is characteristic of alt-NHEJ [4].

**Figure 3:**
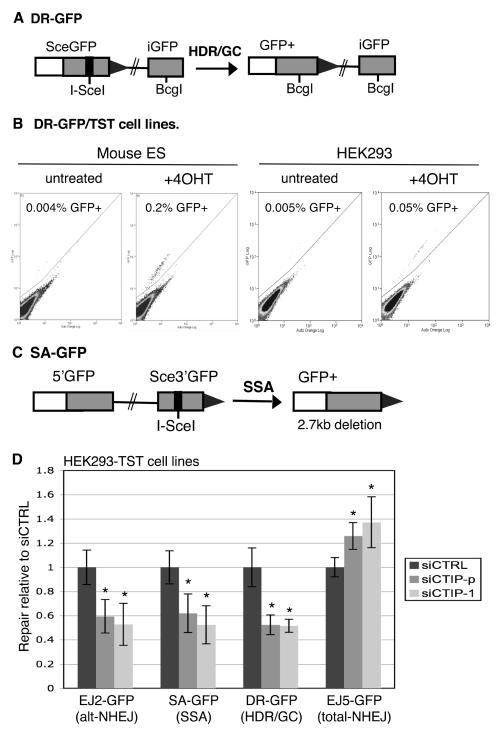
An inducible system for I-SceI in stable cell lines used to show that siRNA-mediated disruption of CtIP affects multiple repair pathways. (A) Shown is the structure of the DR-GFP reporter along with the HDR/GC repair product that results in GFP+ cells, as described previously in ES cells [18]. (B) System for inducible control of I-SceI in stable cell lines. Cell lines were established with ES cells and HEK293 cells that contain the DR-GFP reporter and stable expression of the TAM-I-SceI-TAM (TST) fusion protein. These cell lines were either left untreated, or treated with 4-hydroxytamoxifen (4OHT) for a limited time (8 h for ES, 24 h for HEK293), and analyzed 3 d after starting the treatment. Shown are flow cytometric (FACS) profiles of 10^5^ cells, where green fluorescence is plotted on the *y*-axis and auto orange fluorescence is on the *x*-axis. (C) Shown is the structure of SA-GFP reporter along with the GFP+ product of SSA repair. As discussed previously, HDR/GC associated with crossing over does not likely contribute significantly to this assay [18]. (D) CtIP promotes alt-NHEJ, SSA and HDR, but is dispensable for total-NHEJ. HEK293 cell lines with individual reporters were exposed to control siRNA (siCTRL), a pool of three CtIP-targeting siRNAs (siCTIP-p), or a distinct single CtIP-targeting siRNA (siCTIP-1). Subsequently, I-SceI was activated by 4OHT, and repair was measured as in (B). Shown are repair frequencies relative to the mean value of siCTRL samples treated in parallel. Asterisks denote a statistical difference from siCTRL with the substrates EJ2-GFP, SA-GFP, DR-GFP, and EJ5-GFP for both siCTIP-p (*p*<0.0001, *p* = 0.0012, *p*<0.0001, and *p* = 0.0009, respectively) and siCTIP-1 (*p* = 0.0021, *p* = 0.0002, *p*<0.0001, and *p* = 0.0023, respectively).

From previous studies [4],[6], and the above experiments with EJ5-GFP (Figure 1B and 1C), alt-NHEJ appears to be KU-independent. To investigate this notion further, we compared the efficiency of EJ2-GFP repair in wild-type and *Ku70-/-* ES cells following transfections with an I-SceI expression vector. We found that the *Ku70-/-* cells exhibited a 4-fold increase in the restoration of the *GFP+* gene over wild-type cells, and that this increase was reversed by co-transfection of a KU70 expression vector (Figure 2C). Furthermore, analysis of GFP+ products from *Ku70-/-* showed a similar pattern as wild-type cells, in that the XCM1+ alt-NHEJ product was predominant (Figure 2B). Thus, the alt-NHEJ repair events measured by EJ2-GFP are not only KU-independent, but also appear to be inhibited by KU. In relation to other pathways, KU also suppresses HDR/GC and SSA, as described previously [18], and as confirmed in parallel experiments with EJ2-GFP (Figure 2C, see Figure 3). Given that KU-deficiency can lead to elevated DSB end-processing [19],[20], these results raise the possibility that alt-NHEJ, SSA, and HDR/GC share such end-processing as a common intermediate.

### Inducible System for I-SceI in Stable Cell Lines

To continue to test the above hypothesis, we sought to perform siRNA experiments in HEK293 cells with the DSB reporters. However, we first would like to describe a novel technological approach for such siRNA experiments. In general, use of I-SceI-based reporters for such experiments would require transfection of the siRNA followed by a second transfection of the I-SceI-expression vector. Such serial transfections appear to cause increased toxicity, which can lead to variability between experiments (unpublished observations).

Thus, we have developed a method for inducible activation of I-SceI in stable mammalian cell lines to bypass the need for a second transfection during siRNA experiments. Specifically, we used a mutant form of the estrogen receptor ligand binding domain, where in the absence of 4-hydroxytamoxifen (4OHT), this domain (TAM) appears to restrict access of fused proteins to chromosomes, while addition of 4OHT releases this restriction [21]. We made a series of expression vectors for fusion proteins between the TAM-domain and I-SceI (see Figure S1), and we chose to continue with an expression vector for TAM fused to both ends of I-SceI: TAM-I-SceI-TAM (TST).

We generated stable cell lines expressing the TST fusion protein using a wild-type ES cell line and an HEK293 cell line, each containing an integrated copy of the DR-GFP reporter (see Materials and Methods). Repair of DR-GFP by the HDR/GC pathway results in the restoration of a *GFP* gene (Figure 3A), as previously described in mouse embryonic stem (ES) cells [18]. Following establishment of the TST-expressing cell lines, we analyzed 4OHT-dependent activation of I-SceI, as measured by GFP+ cells. From these experiments, we found low background levels of GFP+ cells from untreated samples, whereas 4OHT treatment resulted in an approximate 50-fold and 10-fold induction of GFP+ cells in ES cells and HEK293 cells, respectively (Figure 3B). Furthermore, we found that the low background levels were stable for at least 4-6 weeks of continuous culture (unpublished data).

To measure not only HDR/GC, but also other repair pathways using this method, we subsequently developed similar TST stable cell lines with HEK293 cells containing the EJ2-GFP, EJ5-GFP, and SA-GFP reporters (see Materials and Methods). The SA-GFP reporter measures SSA (Figure 3C), as previously described in ES cells [18]. As discussed in this previous report, while it is formally possible that HDR/GC associated with crossing-over (CO) could also result in a *GFP+* product from SA-GFP, two lines of evidence strongly suggest that CO events provide a negligible contribution to this assay. For one, multiple independent analyses have shown that CO during DSB repair in mammalian cells occurs at a frequency of less than 1% of the efficiency of the *GFP+* repair events measured by SA-GFP [18],[22],[23]. As well, disruption of strand-exchange factors (BRCA2/RAD51) causes a significant increase in the efficiency of *GFP+* repair of SA-GFP [18], which is inconsistent with a CO mechanism. In summary, we have generated HEK293 cell lines with stable expression of an inducible I-SceI (TST) and four different reporters to measure alt-NHEJ (EJ2-GFP), total-NHEJ (EJ5-GFP), SSA (SA-GFP), and HDR/GC (DR-GFP).

### The End-Processing Factor CtIP Promotes alt-NHEJ, SSA, and HDR/GC, But Is Dispensable for the Absolute Levels of total-NHEJ

As described above, we sought to examine whether DSB end-processing may be a common mechanistic step in alt-NHEJ, SSA, and HDR/GC. For this, we focused on the factor CtIP [24], which is important for processing DSBs into ssDNA, detected as RPA-foci in mammalian cells following DNA damage [25],[26]. Regarding repair pathways, CtIP appears important for HDR/GC in both human cells and *S. pombe*, but is dispensable for plasmid end joining in *S. pombe* [25],[27]. We tested the hypothesis that CtIP in mammalian cells promotes not only HDR/GC, but also alt-NHEJ and SSA.

For this, we performed siRNA knock-down of CtIP in the relevant HEK293 cell lines with individual reporters and stable expression of the inducible I-SceI protein (TST). We knocked-down CtIP levels using two different siRNA reagents: a pool of three siRNA duplexes (siCTIP-p), and a previously described single unique siRNA duplex (siCTIP-1)[25], (see Materials and Methods and Figure S1D). We compared these CtIP-depleted cells to control cells transfected with a non-targeting siRNA (siCTRL). We transfected each set of siRNAs into the HEK293 cell lines 48h prior to induction of I-SceI with 4OHT. The induction with 4OHT continued for 24h, and we assayed repair frequencies (%GFP+ cells) 3d after the start of the 4OHT treatment. We confirmed reduction in CtIP mRNA for both siCTIP-p and siCTIP-1 by RT-PCR of RNA isolated from parallel transfections at the onset of 4OHT addition (see Materials and Methods; unpublished data).

From these experiments, we observed that HDR/GC, alt-NHEJ, and SSA were all significantly reduced in CtIP-depleted cells treated with either siCTIP-p (1.9-fold, 1.7-fold and 1.6-fold, respectively; Figure 3D) or siCTIP-1 (1.9-fold for each pathway; Figure 3D). In contrast, the absolute level of total-NHEJ was slightly increased in CtIP-depleted cells using either siCTIP-p or siCTIP-1 (1.3-fold and 1.4-fold, respectively; Figure 3D). Thus, CtIP appears to promote HDR/GC, alt-NHEJ, and SSA, but is dispensable for total-NHEJ. We suggest that CtIP-mediated DSB end-processing is important to generate ssDNA for the later steps of repair by HDR/GC, alt-NHEJ, and SSA.

### Alt-NHEJ Is Mechanistically Distinct from SSA and HDR/GC During Late Steps of Repair

We also considered how alt-NHEJ might diverge from SSA and HDR/GC at later mechanistic steps. In particular, we addressed how factors important for completion of SSA may influence alt-NHEJ, since both pathways often involve annealing of flanking homology and subsequent processing of non-homologous single-stranded tails.

Regarding SSA, these annealing and processing steps appear to be promoted by RAD52 and ERCC1, since cells deficient in these factors show a decreased level of SSA [18], and also because these factors possess relevant *in vitro* activities. RAD52 can function *in vitro* to directly promote homologous strand annealing, and also to mediate RAD51 function during strand exchange [28]. Though, only the strand annealing activity would be expected to be important for SSA in mammalian cells, since RAD51 appears to inhibit SSA [18]. ERCC1/XPF is a structure-specific endonuclease that catalyzes 5′ excision during nucleotide excision repair [29]. In addition, this complex shows efficient cleavage of 3′ overhangs, which could promote processing of non-homologous single-stranded tails during DSB repair [30]. Furthermore, ERCC1/XPF can form a complex with RAD52, which may suggest that single-strand tail processing and annealing may be coordinated by this complex during repair [31].

**Figure 4:**
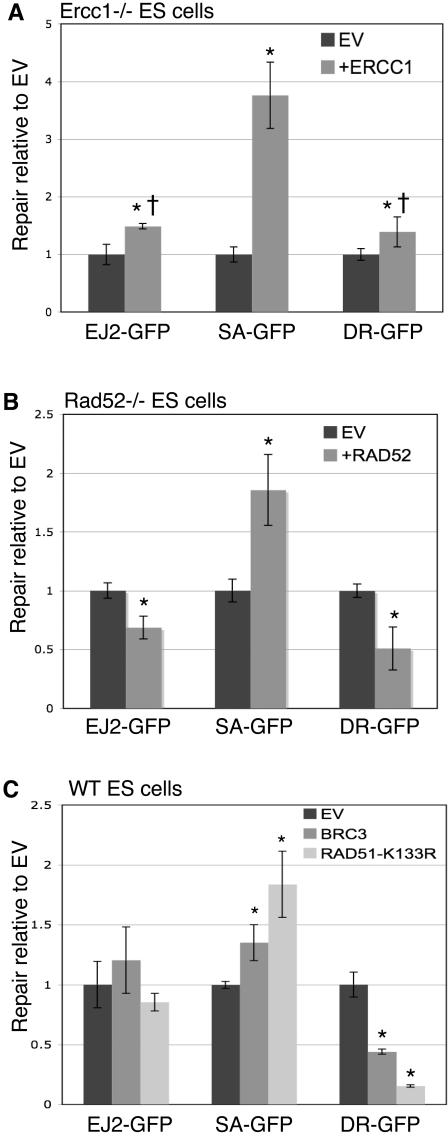
The roles of ERCC1, RAD52, and RAD51 during alt-NHEJ, HDR/GC, and SSA. (A) While ERCC1 significantly promotes SSA, it plays a minor role in HDR/GC and alt-NHEJ. *Ercc1-/-* ES cell lines with EJ2-GFP, SA-GFP, and DR-GFP were transfected with an I-SceI expression vector, along with either an expression vector for ERCC1 or empty vector (EV). Shown are the levels of repair relative to the mean value of a parallel set of EV transfections, which allows a direct comparison of the effect of complementation on the different reporters. Asterisks denote a statistical difference in repair relative to EV (alt-NHEJ and SSA, *p*<0.0001; DR-GFP, *p* = 0.0066), and the dagger denotes a statistical difference in the level of complementation relative to SA-GFP (*p*<0.0001). (B) RAD52 promotes SSA but not HDR/GC or alt-NHEJ. *Rad52-/-* ES cell lines with the reporters shown in (A) were transfected with an I-SceI expression vector, along with either an expression vector for RAD52 or empty vector. Shown are levels of repair as described in (A). Asterisks denote a statistical difference in repair relative to EV (alt-NHEJ, *p* = 0.0003; SA-GFP and DR-GFP, *p*<0.0001). (C) RAD51 promotes HDR/GC, inhibits SSA, and plays no clear role in alt-NHEJ. Wild-type ES cell lines with each of the reporters were cotransfected with an I-SceI expression vector along with either an expression vector for a BRC3 peptide derived from BRCA2, an expression vector for RAD51-K133R, or EV [18]. Shown are levels of repair calculated relative to EV as in (A). Asterisks denote a statistical difference in repair relative to EV (SSA, *p*<0.016; DR-GFP, *p*<0.0008).

To directly examine the role of RAD52 and ERCC1 in alt-NHEJ, we integrated EJ2-GFP into *Rad52-/-* and *Ercc1-/-* ES cells (see Materials and Methods), and determined the fold-change in repair resulting from complementation with the relevant expression vector (i.e. RAD52 or ERCC1). Specifically, we transfected cells with an I-SceI expression vector along with either the relevant complementation vector or empty vector, and then assayed repair three days later as in Figure 2C. As well, previously described *Rad52-/-* and *Ercc1-/-* ES cell lines with DR-GFP and SA-GFP [18] were transfected in parallel. These experiments showed that the efficiency of SSA (SA-GFP) increased upon complementation with each of the relevant expression vectors (3.8-fold for ERCC1, Figure 4A; 1.9-fold for RAD52, Figure 4B). In contrast, the efficiency of alt-NHEJ and HDR/GC only slightly increased by complementation with the expression vector for ERCC1 (1.5-fold, and 1.4-fold, respectively; Figure 4A), and mildly decreased by complementation with RAD52 (1.4-fold reduced, and 2-fold reduced, respectively; Figure 4B). These absolute measurements of alt-NHEJ could include any of the products that result in a *GFP+* gene (see Figure 2A). Notably, while ERCC1 complementation promotes each pathway to some extent, the effect is significantly greater for SSA, as compared to alt-NHEJ and HDR/GC (2.5-fold and 2.7-fold, respectively). These results indicate that alt-NHEJ is mechanistically distinct from SSA, in that this pathway is both less dependent upon ERCC1 and is not promoted by RAD52.

Finally, since the above studies showed several mechanistic similarities between alt-NHEJ and HDR/GC, we next considered a probable mechanistic distinction between these pathways. Namely, we suspected that alt-NHEJ might not require RAD51-mediated strand-exchange. To examine this, we used two dominant negative inhibitors of RAD51: BRC3 and RAD51-K133R. BRC3 is a short peptide derived from BRCA2 that can inhibit RAD51 function [32]. RAD51-K133R is a mutant peptide defective in ATP-hydrolysis that results in hyper-stable strand invasion intermediates [33]. We tested the effect of these peptides on repair of the EJ2-GFP, DR-GFP, and SA-GFP reporters in otherwise wild-type ES cells. For each cell line, we co-transfected the I-SceI expression vector along with vectors expressing either BRC3 or RAD51-K133R, and compared the efficiency of repair relative to cells transfected with I-SceI and empty vector. From these experiments, BRC3 and RAD51-K133R resulted in a 2.3-fold and 6-fold decrease in HDR/GC, respectively, and a 1.4-fold and 1.8-fold increase in SSA, respectively (Figure 4C), which is consistent with previous results [18]. In contrast, from parallel transfections with the EJ2-GFP ES cell line, BRC3 and RAD51-K133R showed no significant effect on alt-NHEJ repair (Figure 4C). Thus, alt-NHEJ is distinct from HDR/GC and SSA, in that it is not affected by disruption of RAD51 function. In summary, alt-NHEJ shows a number of mechanistic distinctions from SSA and HDR/GC during later steps of repair.

## Discussion

**Figure 5:**
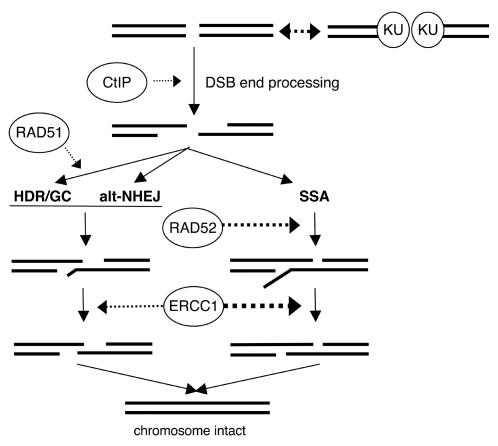
Model for the mechanistic relationships between alt-NHEJ, SSA, and HDR/GC. Individual genetic factors, shown in ovals, are placed in the pathways based on the genetic analysis presented here, and other studies discussed in the text. End processing steps are shown as 5′ to 3′ resection, which need not be the precise mechanism in mammalian cells. The lengths of homologous annealing and 3′ end cleavage are modeled as being less extensive for both alt-NHEJ and HDR/GC relative to SSA.

Chromosomal DSBs can be repaired by a variety of pathways with distinct mechanistic requirements and potentials for mutagenesis. Given the role of mutagenesis during cancer development and treatment, it will be important to understand the mechanistic overlap of these pathways in detail. To this end, we have identified some mechanistic commonalities and differences between three DSB repair pathways: alt-NHEJ, SSA, and HDR/GC (Figure 5).

To begin with, each of these pathways appears to be similarly affected by factors implicated in the control of DSB end-processing, in that they are each suppressed by KU and promoted by CtIP. Such DSB end-processing probably involves 5′ to 3′ resection, as has been directly observed to extend several kilobases in *S. cerevisiae* [34]; however, the precise nature and extent of DSB end-processing has yet to be determined in vertebrate cells. For example, it is possible that ssDNA could be formed via chromatin remodeling followed by unwinding by a DNA helicase. In any case, activation of end-processing likely requires bypassing KU-mediated protection of DSB ends [19],[20],[35]. Such bypass may be initiated by disrupting the binding of KU with DNA. Alternatively, as KU is removed from DSBs, factors could increase the probability that KU-free ends are then processed, for example, by promoting open chromatin structures [36], and/or by activating the end-processing machinery.

CtIP could function via any of these mechanisms during early steps of repair to promote HDR/GC, alt-NHEJ, and SSA. However, its ability to bind to the MRE11 complex and promote its nuclease activity, suggests it may directly promote the end-processing machinery to generate ssDNA [25],[26]. Alternatively, since CtIP is also a transcription factor [37], it could conceivably promote DSB end-processing by opening chromatin or affecting some other upstream process. Notably, CtIP is cell cycle regulated in mammalian cells and in *S. pombe*, showing its highest levels in S-phase through G2/M [27],[38],[39]. Thus, repair pathways that are promoted by CtIP, including alt-NHEJ, might be expected to be more prevalent in these later stages of the cell cycle. In general, further characterization of the nature and mechanism of end-processing in mammalian cells will lead to insight into the role of CtIP in regulating this process during repair. Along these lines, our findings that CtIP promotes repair of both EJ2-GFP and SA-GFP, which involve deletions of 35 nt and 2.7 kb, respectively, suggests that CtIP-mediated DSB end-processing can extend over a relatively wide-range of sizes.

Following DSB end-processing that results in ssDNA as described above, the mechanisms of alt-NHEJ, SSA, and HDR/GC appear to diverge based on how the ssDNA is utilized during repair. For example, such ssDNA could allow either annealing of flanking homology for alt-NHEJ and SSA, or RAD51-mediated strand exchange during HDR/GC. Consistent with this notion, inhibiting RAD51 function disrupts only HDR/GC, such that RAD51 assembly on ssDNA likely commits repair to HDR/GC versus other pathways of repair. Considering the mechanisms of annealing and 3′ end-processing, we have observed that alt-NHEJ is slightly inhibited by RAD52, and is only moderately promoted by ERCC1. In contrast, SSA is significantly promoted by both of these factors. This mechanistic distinction may result from variations in the distance between homologous sequences, the length of the homology, and/or the absolute requirement for homologous annealing. For instance, RAD52 may play a specific role for annealing extensive regions of homology, and hence only promote SSA. This mechanism is supported by *in vitro* studies of RAD52, showing that its preferred binding substrate appears to be long stretches of ssDNA, though some binding to small regions of ssDNA can also be observed [40]. Similarly, the specific role for ERCC1 during SSA could reflect a necessity for this factor in cleaving particularly long 3′ single-stranded tails; however, inconsistent with this model, ERCC1/XPF shows significant cleavage activity on short (15 nt) single stranded tails [30]. Then again, alt-NHEJ may only rarely involve processing of 3′ single-stranded tails, and thus may often involve other intermediate structures that could be cleaved by a different nuclease complex.

Notably, with regard to each of these mechanistic steps of alt-NHEJ, mammalian cells show both similarities and differences with yeast. For instance, our findings with KU/CtIP in mammalian cells are consistent with experiments in *S. cerevisiae* that showed KU-independence [6] and *SAE2*-activation [41] of alt-NHEJ, the latter of which may be relevant to mammalian cells assuming that *SAE2* is a homologue of CtIP [25]. Regarding later steps of alt-NHEJ, the XPF homologue (*RAD10*) in *S. cerevisiae* is critical for this process [6], whereas *RAD52* appears dispensable [42]. Thus, apart from the increased dependence on ERCC1/XPF for alt-NHEJ in yeast, these findings are similar to our results with EJ2-GFP in mammalian cells. In contrast, an *S. pombe* study on alt-NHEJ showed the opposite of the *S. cerevisiae* results, in that XPF (*Rad16*) appears dispensable, and RAD52 (*Rad22*) is important [12]. However, these *S. pombe* experiments were plasmid-based and involved microhomology very close to the end of the DSB. Similarly, a plasmid-based alt-NHEJ assay in *S. cerevisiae* also showed activation of repair by *RAD52* [43]. In general, these distinctions highlight the notion that the mechanism of alt-NHEJ may be distinct between mammalian cells and yeast, but may also be affected by the length of homology, the distance separating the homologous segments, and/or the context of a DSB in a plasmid versus a chromosome.

Reflecting such differences, alt-NHEJ pathways have been categorized using multiple names, each of which reflect certain features of a defined set of repair events: micro-SSA, microhomology-mediated end-joining (MMEJ), KU-independent end-joining, and backup-NHEJ (B-NHEJ) [4]–[12]. While it may be beneficial to find consensus on a particular term, the diversity of terminology also suggest the presence of multiple subclasses of NHEJ events. The predominant event measured by EJ2-GFP, described here as alt-NHEJ, is most similar to MMEJ, in that this product is KU-independent, shows evidence of microhomology at the junction, and results in a deletion. In contrast, other events could be mechanistically more akin to SSA or micro-SSA, with respect to extent of homology, the distance between homologous sequences, and RAD52/ERCC1-dependence. Furthermore, some repair events, while KU-independent, lack evidence of microhomology [4],[9], such that so-called KU-independent NHEJ or B-NHEJ may reflect a larger class of events relative to only MMEJ. Further analysis of the mechanisms of this variety of repair events will continue to clarify the subclasses of NHEJ.

Among these different subclasses of NHEJ, alt-NHEJ/MMEJ appears to play a significant role in the etiology of mutations that arise during cancer development and treatment. For instance, a screen for PARP-inhibitor resistant *BRCA2*-mutant cells revealed a set of reversion mutations that are consistent with alt-NHEJ [14]. Thus, combination of PARP-inhibition and simultaneous disruption of alt-NHEJ may be effective in eliminating PARP-inhibitor resistant cancer cells. As supported by our findings with EJ2-GFP, a target for such therapy may include CtIP [37], whereas disruption of KU-dependent NHEJ pathways would be predicted to be ineffective. Though, PARP has been shown to play a role in plasmid-based NHEJ assays [11], such that it would be important to ensure that alt-NHEJ is targeted separately from PARP function. Similar to the BRCA2 example, tumors deficient in ERCC1 would also be predicted to be relatively proficient at repair of DSBs by alt-NHEJ, which is consistent with the notion that DSB-inducing agents may be less effective on these tumors than interstrand crosslinking agents [44]. Finally, since alt-NHEJ appears to play a significant role in therapy-induced oncogenic chromosomal translocations [13], targeting this pathway, again perhaps via CtIP, may enhance the efficacy of such therapy. In summary, further analysis of the mechanisms and mutagenic potential of individual DSB repair pathways will continue to inform the development of therapeutic approaches to cancer treatment.

## Materials and Methods

### Plasmids and Cell Lines

The expression vector for the fusion protein of TAM-I-SceI-TAM (TST) was generated by PCR amplification of the TAM domain from TAM-CRE [21], and the I-SceI coding sequence from pCBASce, which were cloned in frame into pCAGGS-BSKX [45], as shown in Figure S1. The *EJ2SceGFP* gene (EJ2-GFP) was generated by cloning gcctagggataacagggtaattagatgacaagcc into the XCM1 site of pCAGGS-NZEGFP [46]. *EJ2SceGFP* was then cloned into pim-DR-GFP [47], and downstream of pgk-puro to generate pim-EJ2-GFP and EJ2-GFP-Puro, respectively. For EJ5-GFP, first an I-SceI site was cloned between the AgeI and BclI sites of pim-EJ2-GFP (EJ5sceGFP), and also at the HindIII site of pgk-puro (puroSce). Then, an EcoRI/I-SceI fragment of puroSce was cloned into EJ5SceGFP, followed by cloning an I-SceI site into the EcoRI site of this vector. Pim-EJ5-GFP was then completed by replacement of an EcoRI fragment that was lost in the previous step.

ES cells were cultured as previously described [45], and HEK293 cells (HEK293-A7, New England Biolabs) were cultured according to the directions of the supplier, except we used DMEM high-glucose without phenol red containing Hepes buffer (Invitrogen). HEK293 cells were grown on plates treated with 0.01% poly-lysine (Sigma).

Mouse ES cell lines with DR-GFP and SA-GFP targeted to *hprt* or *Pim1* were described previously [18], [45]–[47]. Pim-EJ2-GFP was used to target the *Pim1* locus of AB2.2 wild-type ES cells [48], and *Ku70-/-* ES cells [49], using methods previously described [45], except targeting was detected by PCR. Pim-DR-GFP, Hprt-SA-GFP, and EJ2-GFP-Puro were randomly integrated into HEK293 cells by electroporation with 1×10^7^ cells suspended in 800 µl PBS in a 0.4 cm cuvette, followed by pulsing the cells at 250 V, 950 µF, and selecting single clones with 3 µg/ml puromycin. Similarly, EJ2-GFP-Puro was randomly integrated into *Ercc1-/-* and *Rad52-/-* ES cells as above, except using electroporation conditions of 680 V and 10 µF. Integration of an intact copy of each randomly integrated reporter was confirmed in single clones by Southern blot analysis with a *GFP* fragment as the probe (data not shown).

Stable cell lines expressing TST were generated by electroporation as described above, except with voltages varying between 200–250V, with 20–30 µg of TST expression plasmid and a selection plasmid. We used two different selection cassettes, with 10 µg of pgk-bsd (gift from Dr. Pentao Liu) for the HEK293 and 5 µg of pmc1neo for the ES cells. Clones were selected in the relevant antibiotic for 6–10d at 400 µg/ml G418 or 5–10mg/ml blasticidin (Invitrogen). Individual selected clones were screened for significant induction of GFP+ cells following 24h treatment with 0.3 µM and 3 µM 4-hydroxytamoxifen (4OHT, dissolved in ethanol, Sigma) for ES and HEK293 cells, respectively.

### Repair Assays

To measure the repair by transient transfection, 2.5×10^4^ cells/cm^2^ were plated and transfected the next day with 0.8 µg/ml of pCBASce mixed with 3.6 µl/ml of Lipofectamine 2000 (Invitrogen) along with a variety of other vectors. The KU and RAD52 expression vectors were added at 0.8 µg/ml, the ERCC1 vector was added at 0.4 µg/ml, the RAD51-K133R vector was added at 0.1 µg/ml, and the BRC3 vector was added at 0.2 µg/ml. For each experiment, an equivalent amount of empty vector (pCAGGS-BSKX) was included in the parallel transfections. Each of these expression vectors have been previously described [18]. GFP positive cells were quantified by flow cytometric analysis (FACS) 3d after transfection on a Cyan ADP (Dako). Amplification of PCR products from sorted GFP+ cells, associated restriction digests, and quantification of bands were performed using the primers KNDRF and KNDRR as previously described for analysis of DR-GFP [50].

To measure repair using the inducible I-SceI protein (TST) in combination with siRNA-mediated inhibition of CtIP, HEK293 cell lines with each of the reporters and stable expression of TST were first plated on 24 well plates at 10^5^ cells/well. The following day, the wells were transfected with 70nM siRNA duplex mixed with 4ul/ml of Lipofectamine 2000 in Optimem (Invitrogen). After 4.5h, transfection complexes were diluted two-fold with media without antibiotics, and 48h after the initiation of transfection, 4OHT was added at 3 µM for 24h. Three days after 4OHT was added, the percentage of GFP+ cells was analyzed by FACS as described above. Knockdown of CtIP levels using the various siRNAs was confirmed by RT-PCR from RNA samples isolated from parallel transfections at the time of 4OHT addition (data not shown). Amplification product was quantified at the threshold cycle by including SYBR green in the PCR reaction and using an iQ5 cycler for real-time analysis at the end of each cycle (BioRad). Products were normalized relative to a primer set directed against actin. Sequences of the siRNAs siCtIP-p (Santa Cruz Biotechnology), and siCtIP-1 [25], and primers for RT-PCR are shown in Figure S1D.

Repair frequencies are the mean of at least three transfections or four 4OHT treatments, and error bars represent the standard deviation from the mean. For some experiments, repair frequencies are shown relative to samples co-transfected with I-SceI and an empty vector (EV). For this calculation, the percentage of GFP+ cells from each sample was divided by the mean value of the EV samples treated in the parallel experiment. Similarly, to calculate the fold-difference in repair between siRNA-treated and control-siRNA treated cells, the percentage of GFP+ cells from each sample was divided by the mean value of control-siRNA samples from the parallel experiment. Statistical analysis was performed using the unpaired *t*-test.

## Supporting Information

Figure S1 **Details of TAM-I-SceI fusion proteins.** (A) Shown is a schematic for control of TAM-I-SceI fusion proteins using the hormone 4OHT. (B) Shown are the primer sequences used to generate expression vectors for TAM-I-SceI fusions from the parent vectors TAM-CRE, along with pCBASce and pCAGGS-BSKX. For TAM-I-SceI (TS): a PCR product of TAM-CRE, using Scetam1 and Scetam7, was cloned into EcoRI/SalI sites of pCAGGS-BSKX, followed by insertion of a BbsI/AvrII fragment of pCBASce. For SceTAM (ST), a PCR product of pCBASce, using Scetam3 and Scetam4, was cloned into EcoRI/BglII sites of pCAGGS-BSKX, followed by insertion into the BglII/XhoI sites of this vector with a PCR product of TAM-CRE using Scetam5 and Scetam8 digested with BamHI/SalI. For TamSceTam (TST): a PCR product of TAM-CRE, using Scetam1 and Scetam7, was cloned into EcoRI/BbsI sites of ST. (C) We tested each of the ST, TS, and TST plasmids by transient transfection into the DR-GFP ES cell line, followed by treatment with 4OHT for 24 h, or untreated. I-SceI activity is measured by induction of HDR/GC, as determined 3 d after the 4OHT treatment. In these experiments, we found that each of the plasmids conferred approximately equivalent I-SceI activity in the presence of 4OHT, while the TST fusion showed the lowest background activity in the absence of 4OHT. (D) Shown are the relevant sequences for the CtIP siRNA experiments, as described in Materials and Methods.
(0.59 MB TIF)

Table S1 **Repair junctions for EJ2-GFP.** PCR products shown in Figure 1C from ES cells were cloned into the PCR2.1 vector (Invitrogen) according to the manufacturer's instructions, and 12 individual clones with detectable inserts were sequenced using the M13F primer. Shown is the sequence surrounding the two I-SceI sites (bold) in the parental EJ5-GFP reporter, along with repair products from sorted GFP+ cells. Products that were identified in multiple independent clones are noted in parentheses. Microhomology found at or near the junctions is underlined, and the length of microhomology is noted. The sizes of the deletions relative to the I-SceI+ product are also shown, starting from the 3′ end of the coding strand (shown as ATAA/ in the I-SceI+ product).
(0.04 MB DOC)

Table S2 **Repair junctions for EJ2-GFP.** PCR products shown in Figure 2B from ES cells were cloned into the PCR2.1 vector (Invitrogen) according to the manufacturer's instructions, and individual clones with detectable inserts were sequenced using the M13F primer. Shown is the sequence surrounding the I-SceI site (bold) in the parental EJ2-GFP reporter, along with various repair products from sorted GFP+ cells. The sequence of the XCM1+ product was confirmed in a clone generated from the uncut PCR product. The sequence of the 23 nt deletion product was found in 3 clones from the XCM1-resistant PCR product, where the junction is marked by a hyphen for clarity. Regarding the larger deletions, 7 clones in total were sequenced, and one product was found twice, as noted in the parentheses. Microhomology found at or near the junctions is underlined, and the length of microhomology is noted, where a discontinuous tract of homology is noted as dis. The sizes of the deletions from the I-SceI cut site are also shown, starting from the 3′ end of the coding strand (shown as ATAA/ in the parent reporter).
(0.04 MB DOC)
