# Supplementary figures and images for: Alternative-NHEJ Is a Mechanistically Distinct Pathway of Mammalian Chromosome Break Repair
Source: PLoS Genet. 2008 Jun 27;4(6):e1000110. doi: 10.1371/journal.pgen.1000110 (PMC2430616; doi:10.1371/journal.pgen.1000110)

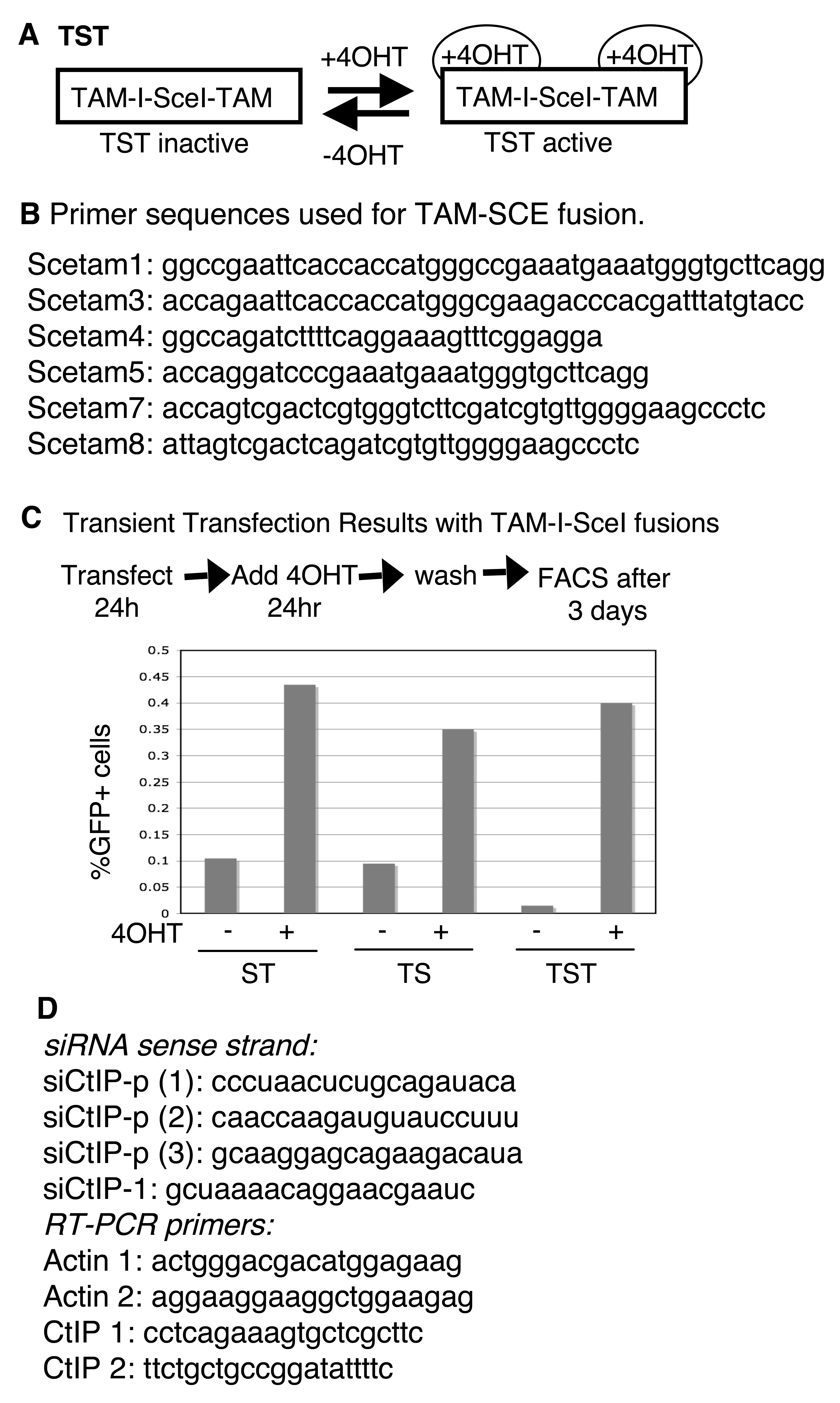

Supplement: Figure S1 — Details of TAM-I-SceI fusion proteins. (A) Shown is a schematic for control of TAM-I-SceI fusion proteins using the hormone 4OHT. (B) Shown are the primer sequences used to generate expression vectors for TAM-I-SceI fusions from the parent vectors TAM-CRE, along with pCBASce and pCAGGS-BSKX. For TAM-I-SceI (TS): a PCR product of TAM-CRE, using Scetam1 and Scetam7, was cloned into EcoRI/SalI sites of pCAGGS-BSKX, followed by insertion of a BbsI/AvrII fragment of pCBASce. For SceTAM (ST), a PCR product of pCBASce, using Scetam3 and Scetam4, was cloned into EcoRI/BglII sites of pCAGGS-BSKX, followed by insertion into the BglII/XhoI sites of this vector with a PCR product of TAM-CRE using Scetam5 and Scetam8 digested with BamHI/SalI. For TamSceTam (TST): a PCR product of TAM-CRE, using Scetam1 and Scetam7, was cloned into EcoRI/BbsI sites of ST. (C) We tested each of the ST, TS, and TST plasmids by transient transfection into the DR-GFP ES cell line, followed by treatment with 4OHT for 24 h, or untreated. I-SceI activity is measured by induction of HDR/GC, as determined 3 d after the 4OHT treatment. In these experiments, we found that each of the plasmids conferred approximately equivalent I-SceI activity in the presence of 4OHT, while the TST fusion showed the lowest background activity in the absence of 4OHT. (D) Shown are the relevant sequences for the CtIP siRNA experiments, as described in Materials and Methods. (0.59 MB TIF) [file pgen.1000110.s001.tif]
