# Supplementary material for: Alternative-NHEJ Is a Mechanistically Distinct Pathway of Mammalian Chromosome Break Repair
Source: PLoS Genet. 2008 Jun 27;4(6):e1000110. doi: 10.1371/journal.pgen.1000110 (PMC2430616; doi:10.1371/journal.pgen.1000110)
Supplement: Table S1 — Repair junctions for EJ2-GFP. PCR products shown in Figure 1C from ES cells were cloned into the PCR2.1 vector (Invitrogen) according to the manufacturer's instructions, and 12 individual clones with detectable inserts were sequenced using the M13F primer. Shown is the sequence surrounding the two I-SceI sites (bold) in the parental EJ5-GFP reporter, along with repair products from sorted GFP+ cells. Products that were identified in multiple independent clones are noted in parentheses. Microhomology found at or near the junctions is underlined, and the length of microhomology is noted. The sizes of the deletions relative to the I-SceI+ product are also shown, starting from the 3′ end of the coding strand (shown as ATAA/ in the I-SceI+ product). (0.04 MB DOC) [file pgen.1000110.s002.doc]

| **Clone** | **Sequence** | **5’ del** | **3’ del** | **Micro-homology** |
| --- | --- | --- | --- | --- |
| **EJ5-GFP** | AGAATTC**TAGGGATAA/CAGGGTAAT..(puro)..** **TAGGGATAA/CAGGGTAAT**GGATCC | N/A | N/A | N/A |
| **Major GFP+ Product** |  |  |  |  |
| I-SceI+ | AGAATTC**TAGGGATAA/CAGGGTAAT**GGATCC | N/A | N/A. | 4 nt./3’ end |
| **Other GFP+ Products** (12) |  |  |  |  |
| 8 nt. del (1) | AGAATTC**TAGGGATAAT**GGATCC | 0 nt. | 8 nt. | 3 nt. |
| 9 nt. del (6) | AGAATTC**TAGGGTAAT**GGATCC | 8 nt. | 1 nt. | 4nt.. |
| 11 nt. del (2) | AGAATTC**TAGGGAT**GGATCC | 2 nt. | 9 nt. | 2nt. |
| 11 nt. del (1) | A**ATAA/CAGGGTAAT**GGATCC | 15 nt. | **4nt. fill** | None |
| 15 nt. del (1) | AGAATTC**TAGGGAT**cca | 2 nt. | 13 nt. | 2 nt. |
| 27 nt. del (1) | cat**CAGGGTAAT**GGATCC | 27 nt. | 0 nt. | 2 nt. |
