# Supplementary material for: Alternative-NHEJ Is a Mechanistically Distinct Pathway of Mammalian Chromosome Break Repair
Source: PLoS Genet. 2008 Jun 27;4(6):e1000110. doi: 10.1371/journal.pgen.1000110 (PMC2430616; doi:10.1371/journal.pgen.1000110)
Supplement: Table S2 — Repair junctions for EJ2-GFP. PCR products shown in Figure 2B from ES cells were cloned into the PCR2.1 vector (Invitrogen) according to the manufacturer's instructions, and individual clones with detectable inserts were sequenced using the M13F primer. Shown is the sequence surrounding the I-SceI site (bold) in the parental EJ2-GFP reporter, along with various repair products from sorted GFP+ cells. The sequence of the XCM1+ product was confirmed in a clone generated from the uncut PCR product. The sequence of the 23 nt deletion product was found in 3 clones from the XCM1-resistant PCR product, where the junction is marked by a hyphen for clarity. Regarding the larger deletions, 7 clones in total were sequenced, and one product was found twice, as noted in the parentheses. Microhomology found at or near the junctions is underlined, and the length of microhomology is noted, where a discontinuous tract of homology is noted as dis. The sizes of the deletions from the I-SceI cut site are also shown, starting from the 3′ end of the coding strand (shown as ATAA/ in the parent reporter). (0.04 MB DOC) [file pgen.1000110.s003.doc]

| **Clone** | **Sequence** | **5’ del** | **3’ del** | **Micro-homology** |
| --- | --- | --- | --- | --- |
| **EJ2-GFP** | TTCGCCAAGCCCGCC**TAGGGATAA/CAGGGTAAT**TAGATGACAAGCCCGGCG | N/A | N/A | N/A |
| **Major GFP+ Product** |  |  |  |  |
| 35 nt. del XCM1+ | TTCGCCAAGCCCGGCG | 19 nt. | 16 nt. | 8 nt. |
| **Minor GFP+ Products** |  |  |  |  |
| *XCM1-r product (3):*  23 nt. del (3, all) | TTCGCCAAGCCCGC-TGACAAGCCCGGCG | 10 nt. | 13 nt. | none. |
| *Small products (7):*  146 nt. del (1) | ccggcaccGACAAGCCCGGCG | 132 nt. | 14 nt. | 2+6 nt. dis. |
| 310 nt. del (1) | gaattcAGATGACAAGCCCGGCG | 300 nt. | 10 nt. | 4 nt. |
| 325 nt. del (1) | catcatttTGACAAGCCCGGCG | 312 nt. | 13 nt. | 2+4 nt. dis. |
| 329 nt. del (1) | tcattttgGCCCGGCG | 310 nt. | 19 nt. | 2 nt. |
| 338 nt. del (2) | gtgct**GGGTAA**TTAGATGACAAGCCCGGCG | 336 nt. | 2 nt. | 2 nt. |
| 362 nt. del (1) | gtacgga**AGGGTAAT**TAGATGACAAGCCCGGCG | 361 nt. | 1 nt. | 2 nt. |
